# Perivascular epithelioid cell tumor (PEComa) of the uterine cervix associated with intraabdominal "PEComatosis": A clinicopathological study with comparative genomic hybridization analysis

**DOI:** 10.1186/1477-7819-2-35

**Published:** 2004-10-19

**Authors:** Oluwole Fadare, Vinita Parkash, Yesim Yilmaz, M Rajan Mariappan, Linglei Ma, Denise Hileeto, Mazin B Qumsiyeh, Pei Hui

**Affiliations:** 1 Department of Pathology, Yale University School of Medicine, New Haven, CT, USA; 2 Department of Laboratory Medicine Yale University School of Medicine, New Haven, CT, USA; 3 Department of Genetics, Yale University School of Medicine, New Haven, CT, USA; 4 Department of Pathology, Hospital of St Raphael, New Haven, CT, USA; 5 Department of Pediatrics, Louisiana State University Health Sciences Center, New Orleans, LA, USA; 6 Department of Pathology, Stanford University, Stanford, CA, USA; 7 Department of Pathology, New York University, New York, NY, USA

## Abstract

**Background:**

The World Health Organization recently recognized a family of neoplasms showing at least partial morphological or immunohistochemical evidence of a putative *perivascular epithelioid cell *(PEC) differentiation. These tumors include angiomyolipoma (AML), clear cell "sugar" tumors of the lung (CCST), lymphangioleiomyomatosis (LAM), clear cell myomelanocytic tumors of the falciform ligament and distinctive clear cell tumors at various other anatomic sites.

**Case presentation & methods:**

A 41-year old gravida-1 para-1 with tuberous sclerosis presented with an incidentally identified 2.2 cm mass. The morphology and immunohistochemical profile was consistent with PEComa. Distinct aggregates of HMB-45 epithelioid cells were present in an occasionally distinctive perivascular distribution in the myometrium, small bowel lamina propria and ovarian hila. These distinctive aggregates, for which we propose the designation "PEComatosis" based on their intraabdominal distribution, did not display cytological atypia, mitotic activity or necrosis. CGH and DNA ploidy analysis showed a balanced chromosomal profile and diploid nuclei, respectively. There was no recurrence or metastases at 35 months' follow-up. Fifty-one previously reported cases of non-AML, LAM and CCST PEComas [perivascular epithelioid cell tumors- not otherwise specified (PEComa-NOS)] are reviewed.

**Conclusions:**

The lesions may be a reflection of tumor multicentricity, in which each may be a potential nidus for the development of future more well-developed tumors. Alternatively, they may be a manifestation of a poorly understood "field effect", in which there is an increased propensity to develop tumors of this type throughout the abdomen. Finally, and least likely in our opinion, they may represent tumor spread from its primary site.

## Background

Perivascular epithelioid cell tumors (PEComa) have been the subject of abundant discussion in the medical literature over the past decade [1-47]. Morphologic and immunophenotypic similarities between some constituent cells of renal angiomyolipomas (AML) and those of a case of clear cell "sugar" tumor of the lung (CCST) were initially noted in 1991 [33]. One year later, Bonetti *et al *[4], formally proposed the concept of "perivascular epithelioid cell" (PEC), a then provisional term meant to describe the epithelioid cells that characterize, at least in part, the aforementioned lesions. Characteristics of PEC (which does not have a normal anatomic homologue) include co-expression for melanocytic and muscle markers, epithelioid to spindle cellular shapes with ample clear to eosinophilic cytoplasm, and at least in some cases, arrangement around blood vessels [2]. Ultrastructurally, structures interpreted as melanosomes and premelanosomes have been demonstrated in some tumors composed of PECs [14,18,31,38], but not in others [12,19,20,28,41]; an additional case showed macroscopic pigmentation [1]. In 1994, based on the morphologic and immunophenotypic distinctiveness of PECs, in addition to the fact that similar cells had been described in some other tumors, Bonetti *et al *proposed the concept of a family of lesions sharing this cellular phenotype, including CCST, AML, and lymphangioleiomyomatosis (LAM) [5]. The term "PEComa" was introduced by Zamboni *et al *[42] in 1996 as synonym for this family of tumors.

Over the past decade, PEC and tumors composed of them have engendered significant discussions and controversies with respect to their very existence as a clinico-pathological entity, their histogenesis, pathogenesis, and nomenclature [2-6,16,17,25,26,32,33,35,39,40]. Nonetheless, in 2002 and 2003, two monographs published under the auspices of the World Health Organization (WHO) recognized a family of neoplasms with perivascular epithelioid cell differentiation and accepted the designation "PEComa" [13,21]. In the WHO *soft tissue *volume, PEComas are defined as "mesenchymal tumors composed of histologically and immunohistochemically distinctive perivascular epithelioid cells" [13]. Members of the PEComa family that were recognized include AML, CCST, lymphangioleiomyomatosis (LAM), clear cell myomelanocytic tumor (CCMMT) of the falciform ligament/ ligamentum teres and a heterogeneous group of other "unusual clear cell tumors" at various anatomic sites [13]. The latter group includes tumors that have been reported under varying designations, such as abdominopelvic sarcoma of perivascular epithelioid cells [6], primary extrapulmonary sugar tumor (PEST) [38], clear cell myomelanocytic tumors of the skin [7] and thigh [15], and simply *PEComa *of various anatomic sites [1,9,12,19,24,27,28,31,40,41,45,46]; these, in addition to CCMMT of the falciform ligament [14] will henceforth be referred to as PEComa not otherwise specified (PEComa NOS). This descriptive designation, as used in this report, excludes the well-established entities LAM, CCST of the lungs and all variants of AML. Most of the reported cases of PEComa NOS have been tumors located in the uterine corpus (21/51; 41%); however, consequent to the publication of the WHO monographs, there has been a recent noticeable increase in the number of reported cases of PEComa NOS, with almost 70% of all cases reported between 2001 and 2004 [1,6-8,10-12,15,19,20,22,24,27,28,31,38,40,41,43-46](additional file 1). Concurrently, there has been an increase in the diversity of anatomic sites from which they reportedly arose, such that, it now appears that these tumors may potentially arise from any anatomic location. The morphologic and immunophenotypic spectrum as well as exclusion/inclusion criteria of PEComa NOS are not well-defined, and pathologic parameters with prognostic value have yet to be elucidated. In addition, there is a striking scarcity of information on the molecular and cytogenetic data of these lesions, probably due to their rarity. In this report, we present an example of a PEComa NOS of the uterine cervix, with the following objectives: 1) to document the uterine cervix as another potential site for a PEComa NOS, 2) based on the presence of tumorlets outside of their primary site, to analyze morphologic criteria predictive of clinical behavior of PEComa NOS, 3) to present CGH and ploidy analysis of PEComa NOS, and 4) to present another example of a PEComa NOS occurring in a tuberous sclerosis patient, an association which has been documented only twice previously [6,40]).

## Case presentation

**Figure 1:**
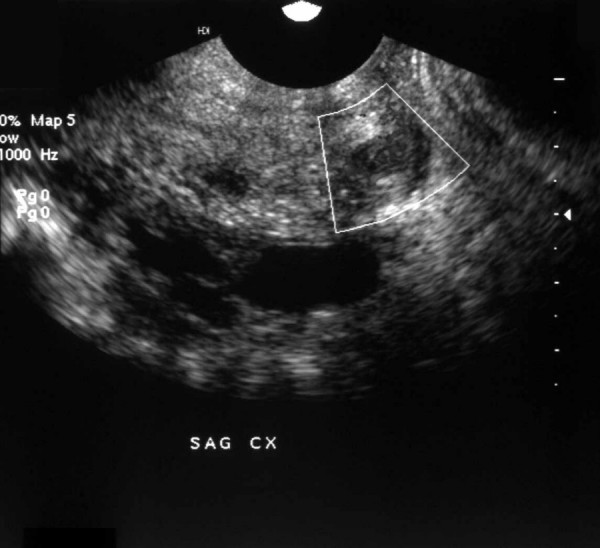
Ultrasonographic appearance of the cervical mass showing it to be deceptively circumscribed

In October 2001, a left pelvic adnexal mass was palpated during a routine physical examination of a 41-year-old gravida-1 para-1 Caucasian female. The patient's past medical history was significant for tuberous sclerosis (diagnosed previously based on seizures, radiographic evidence suggestive of lymphangioleiomyomatosis, cutaneous hypopigmented macules and bilateral cystic renal disease that culminated in end-stage renal disease in 1989). A transvaginal ultrasound showed a heterogeneous complex adnexal mass whose size was estimated at 7.0 × 4.8 cm. Also noted was a well-defined lesion (<1.0 cm) in the uterine cervix (figure 1); the clinical impression of the latter lesion was a leiomyoma. CA-125 (13 U/ml) and CA19-9 (21 U/ml) levels were within normal limits. The decision was made to resect the adnexal mass and in November 2001, the patient underwent a total abdominal hysterectomy with bilateral salpingo-oophorectomy. The surgical procedure was complicated by severe intra abdominal adhesions (secondary to long-term peritoneal dialysis), the lyses of which resulted in two inadvertent nicks to small bowel segments that necessitated the excision of those segments. The patient did not receive any adjuvant or neoadjuvant therapy, and she remains alive with no evidence of recurrent or metastatic disease after 35 months of close surveillance.

### Sample preparation

Standard representative sections, including the entirety of the uterine cervix were processed routinely for microscopic examination. Sections were fixed in 10% neutral buffered formalin, embedded in paraffin and stained with hematoxylin and eosin. Selected sections were stained for Periodic Acid Schiff (PAS) with and without diastase pre-digestion. The immunohistochemical profile of the tumor was evaluated on 4μ thick, formalin-fixed, deparaffinized sections using a DAKO Autostainer (Carpinteria, CA, USA) based on the avidin-biotin-peroxidase complex. Specifications for the various immunohistochemical stains that were utilized are listed in table 1. The extent and intensity of the immunoreactivity for each antibody was scored semi-quantitatively on a 1+(+) to 4+(++++) scale representing increasing staining extent and intensity. Labeling index for Ki-67 was calculated by assessing at least 4000 cells and determining the percentage showing unequivocal nuclear staining. In our literature review, the significance of the differences between the group means of two continuous variables (patient age and sizes of lesions) was determined using the student's t-test (Excel^®^, Microsoft Inc, Redmond, WA). DNA ploidy analysis was performed on isolated tumor nuclei according to standard procedures. Comparative genomic hybridization [48] was performed on tumor tissue samples from the cervical mass as previously described [49].

**Table 1:** Immunohistochemistry: antibody specifications and results

**Antibody**	**Clone**	**Dilution**	**Antigen retrieval method**	**Vendor**	Results*	
					
					**Spindle cells**	**Epithelioid cells**
HMB-45	HMB-45	1:200	None	DakoCytomation Carpinteria, CA, USA	+++	+++
Vimentin	V9	1:5	None	Ventana, Tucson AZ, USA	+++	+
Desmin	D33	1:250	Trypsin	Ventana	+++	+
Progesterone receptor (PR)	PGR 636	1:2	Steam¶	DakoCytomation	++	++
Melan-A	A103	1:40	Steam	DakoCytomation	+++	+++
Estrogen receptor (ER)	1D5	1:2	Steam	DakoCytomation	-	-
Caldesmon	H-CD	1:100	Steam	DakoCytomation	+	-
Smooth muscle actin (SMA)	1A4	Neat	None	Sigma, St Louis, MO, USA	+++	++
Keratin	AE1/AE3	1:1200	Trypsin	Chemicon Int, Temecula, CA	-	-
S100	Polyclonal	1:2	Pronase	DakoCytomation	+	-
Ki-67 (LI)^¶^	MIB-1	1:300	Steam	DakoCytomation	+(<1%)	+(<1%)
CD68	PG-M1	1:4	Steam	DakoCytomation	-	-
CD117	Polyclonal	1:200	Steam	DakoCytomation	-	-
EMA	E29	1:1000	None	DakoCytomation	-	-
p53	D07	1:3200	Steam	DakoCytomation	-	-

¶ Heat-induced epitope retrieval, 20 minutes in 10 mM citrate buffer in steam chamber, 20 minutes cooling down period.

¶ LI: Labeling Index

*Semi-quantitative scoring of combined extent and intensity of staining (+ to ++++)

### Pathological findings

**Figure 2:**
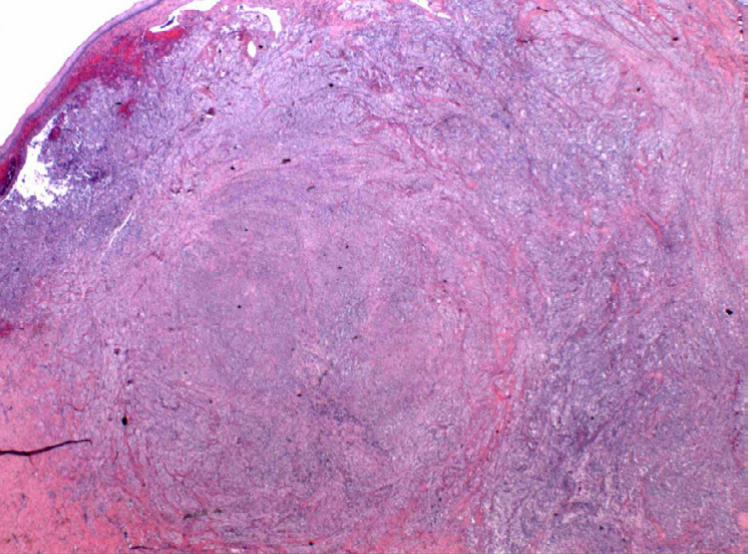
Photomicrograph of panoramic view of the cervical mass showing a central circular "core"

**Figure 3:**
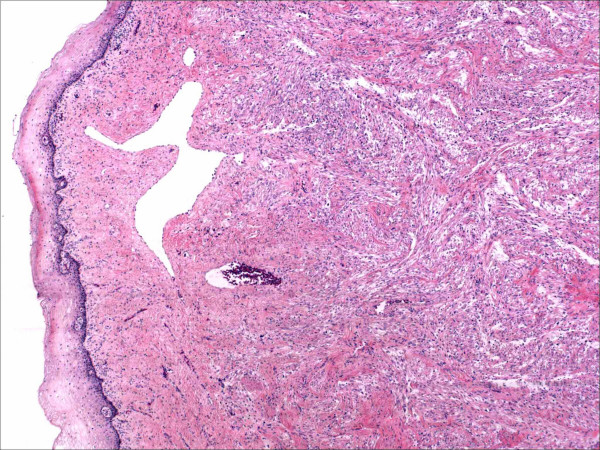
At the tumor's advancing edge, it merges almost imperceptibly with the sub-ectocervical stroma

**Figure 4:**
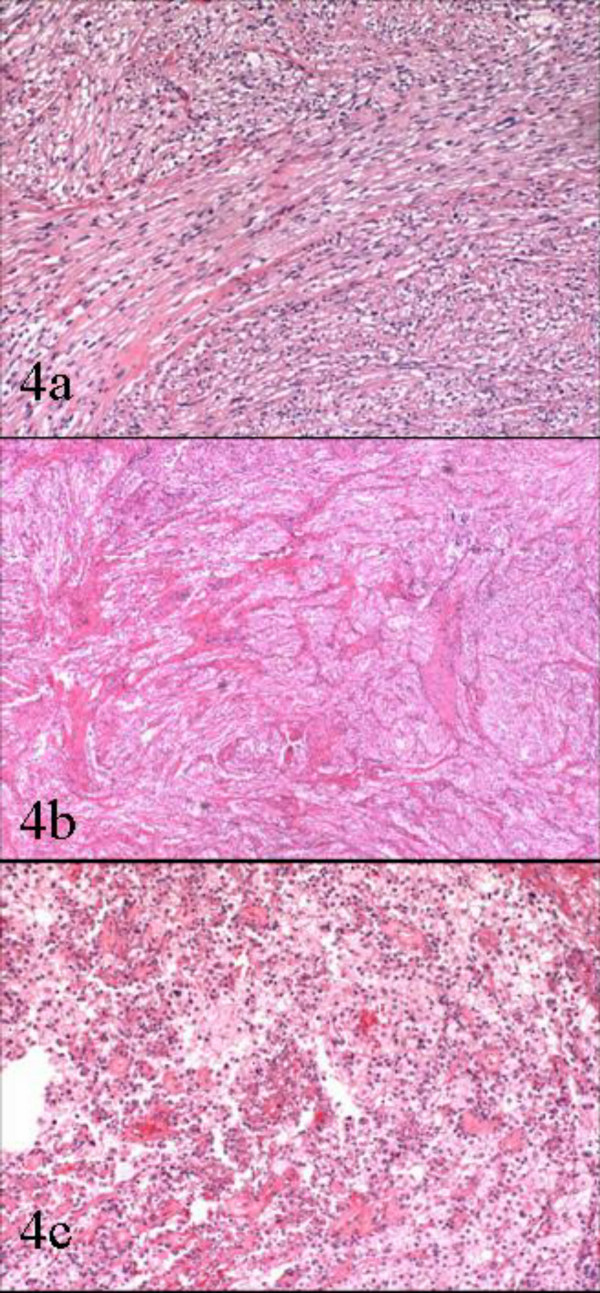
The cervical mass displayed a morphologic spectrum from purely spindle, smooth-muscle-like areas (figure 4a) to transitional areas composed of spindle cells with more clear cytoplasm (figure 4b) to overtly epithelioid areas with abundant, clear cytoplasm, which occasionally displayed a pseudo-alveolar appearance (figure 4c)

**Figure 5:**
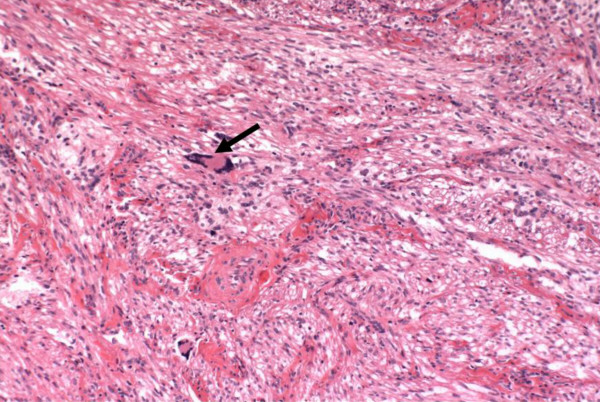
Occasional cells showed degenerative multinucleation with a "smudged" chromatin nuclear pattern consistent with degenerative atypia

**Figure 6:**
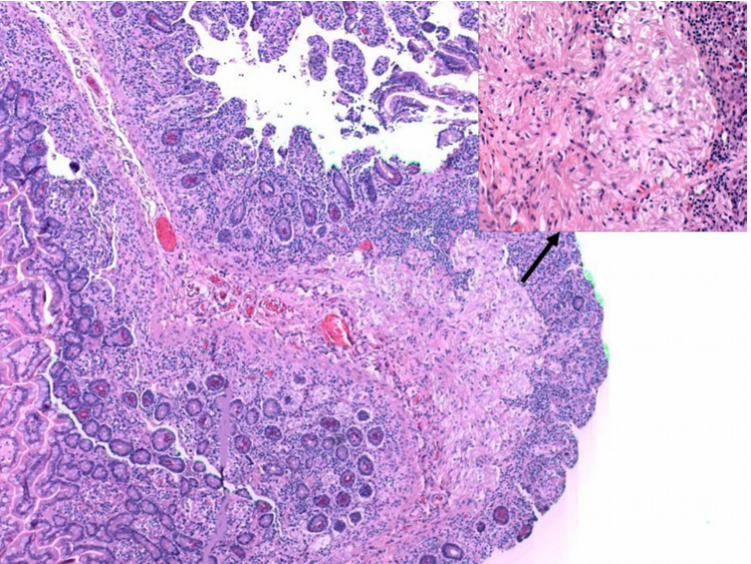
Aggregates of spindle to polygonal cells with eosinophillic to clear cytoplasm was present in the lamina propria of the excised small bowel segments. These aggregates were HMB-45-positive.

**Figure 7:**
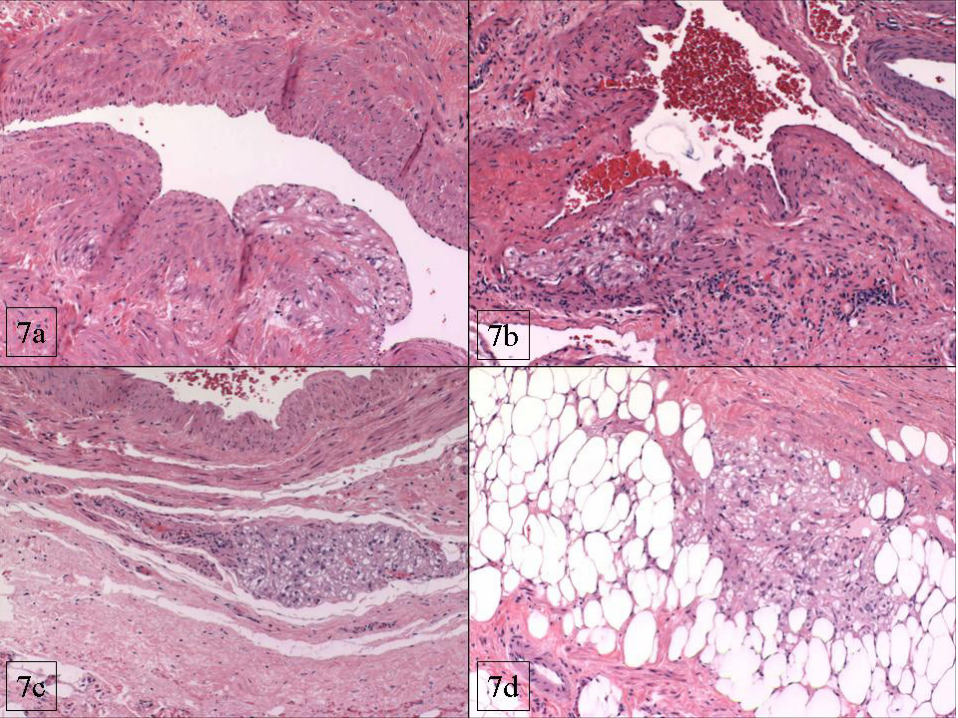
In the ovarian hila, similar tumor aggregates were associated with vascular structures in a subendothelial (figure 7A), intramedial (Figure 7B), or para-adventitial (Figure figrure 7C ) pattern, but were also present freely in the hilar fat (Figure 7D).

**Figure 8:**
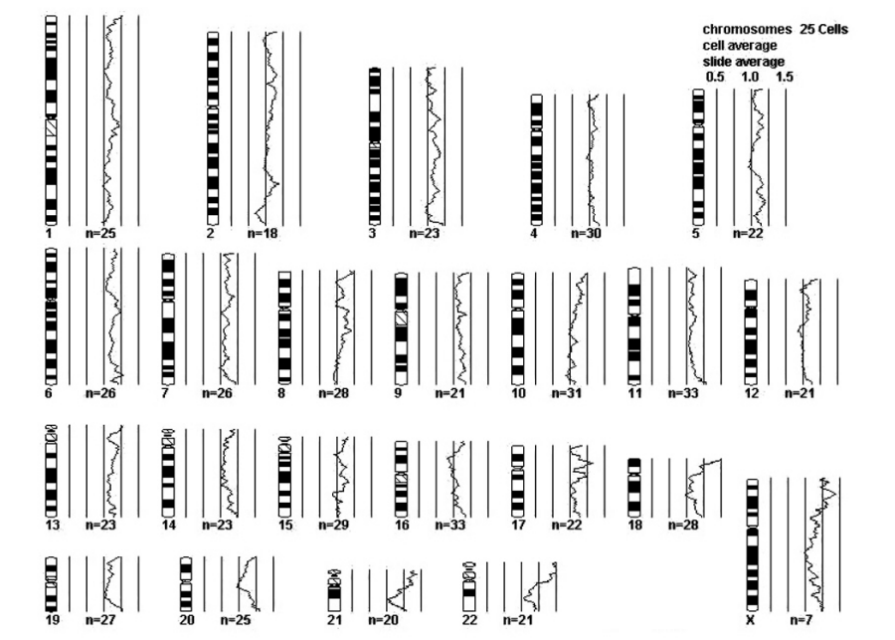
Comparative genomic hybridization showing a balanced chromosomal profile

**Figure 9:**
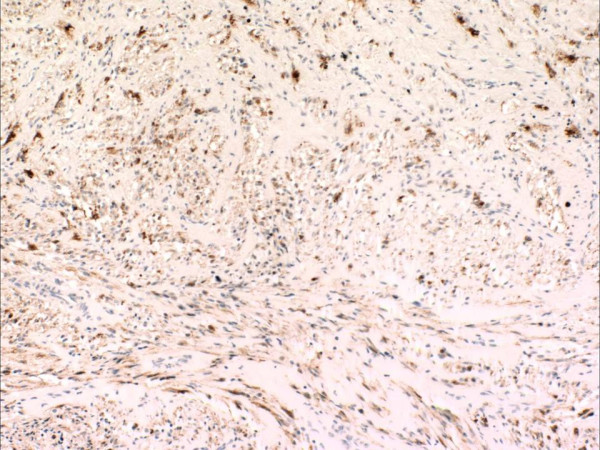
All components of the tumor were HMB-45-positive

Gross and microscopic assessment of the left adnexal mass showed it to be a 7 cm hemorrhagic cyst devoid of any specific lining and involving the ovarian parenchyma. For the uterine cervical mass, a distinct lesion was not grossly appreciated. The ectocervical and endocervical surfaces and the endometrial cavity were described as unremarkable. Microscopically, the cervical mass was unencapsulated but possessed a deceptively circumscribed appearance at scanning magnification, attributable to the architectural homogeneity of its "core" (Figure 2). However, the peripheral regions of the tumor showed a significant degree of infiltration. The tumor's maximal dimension was estimated at 2.2 cm, extending from just below the ectocervical basement membrane (Figure 3) and extending proximally to the lower uterine segment and attaining 2 cm in depth (the peripheral limits of the tumor were at least 1 cm from the parametrial margins). The aforementioned central "core" (1 cm) was probably responsible for its radiographic appearance and consisted of fascicles of spindle cells with a smooth muscle appearance (Figure 4a). The spindle cells displayed bland nuclei with regularly distributed chromatin and only rarely conspicuous nucleoli. Towards the periphery, the spindle cells displayed increasingly PAS+, diastase sensitive cytoplasm (Figure 4b), although occasional cells displayed dense eosinophilia. At its most peripheral regions, the tumor was composed predominantly of solid sheets of large epithelioid cells with bland nuclear features, abundant clear cytoplasm, and well-defined cytoplasmic membranes. Although predominantly solid in the epithelioid regions, a pseudo-alveolar pattern was also evident (Figure 4c). At the most proximal regions near the lower uterine segment, the tumor appeared to be "invading" in single cells in a hyalinized stroma. The nuclei of the epithelioid cells showed a mild to moderate degree of nuclear atypia, manifested mostly as nucleomegaly and irregularity of nuclear membranes in the absence of hyperchromasia. Rare cells displayed bizarrely enlarged nuclei and multinucleation with a "smudged" chromatin pattern consistent with degenerative atypia (Figure 5). Also identified in these regions were CD68+ foamy histiocytes mostly in single cells but occasionally in aggregates especially around the endocervical glands. No tumor necrosis was identified and mitotic figures were extremely sparse (<1/50 HPF). Small arching sinusoidal vessels were prominent throughout the tumor, but no large malformed vascular profiles were present. Pigment-laden cells and adipocytes were not present in the cervical mass. Small bowel segments measuring 21 cm in total length were also processed. Grossly, irregular areas of transmural thickening were noted. Microscopically, aggregates of epithelioid cells with more eosinophillic cytoplasm and vacuolated cytoplasm were noted in the lamina propria in two out of twelve sections (Figure 6). In the both ovaries, similar aggregates of cells were present in a distinctive perivascular location in the hilar regions. These aggregates were either subendothelial (predominantly), adventitially attached to affected vessels, or present as free aggregates in the perihilar fat (Figure 7). Each measured less than 1 mm in maximum dimension. No intraluminal tumor cells were seen. CGH and DNA ploidy analysis of the cervical mass showed a balanced chromosomal profile (figure 8) and diploid nuclei, respectively. Immunohistochemically, both the epithelioid cells and spindle cells stained diffusely with HMB-45 (Figure 9) and Melan-A in a cytoplasmic pattern at all sites (cervix, ovary, bowel). Scattered spindle cells showed unequivocal immunoreactivity for S100 while the epithelioid cells were negative. Both components showed at least focal immunoreactivity for muscle markers: smooth muscle actin and desmin with the spindle cells predominating both in the quantity of cell stained and the intensity of staining where positive. The complete immunohistochemical profile of the tumor is shown in table 1.

## Result of literature review

Of the 51 cases of PEComa NOS that have been documented in the literature [1,6-8,10-12,14,15,19,20,22-25,27,28,31,32,34,37,38,40-46], 90% (46/51) developed in females and 41% (21/51) were described in the uterine corpus. Follow-up information was unavailable (n = 7) or too recent at the time of the report (n = 4) in 22% of cases [7,10,14,15,34,40,43,46]. One patient whose tumor was primary in the left atrium died postoperatively of "cardiac failure thought to be due to nontumorous coronary artery thromoboemboli" [37]. Two cases were excluded from the following analysis (44, 45) based on insufficient morphologic information (44) or inconsistence with our analytic paradigm (45). Of the remaining 37 cases (additional file 2), the associated tumors behaved in a benign fashion in 25 cases (68%). These tumors were confined to their respective primary sites and showed no evidence of recurrence or metastases with follow-up periods that ranged from 6 weeks to 22 years, and will hereafter be referred to as the "benign cases" [1,6,8,14,20,23,25,27,32,37,38,40,42,44]. However in twelve cases, the tumors recurred after apparently complete surgical resections, were either metastatic at presentation or metastasized after long periods. Four of these cases were ultimately fatal [6,19,22,28], and all 12 will hereafter be referred to as the "non-benign cases" [6,11,12,14,19,22,24,28,31,41].

Clinical parameters were not particularly discriminatory between the two groups: the average patient age of the benign cases (37 years) was not significantly different from that of the non-benign cases (43 years) [p = 0.56] and there was a diversity of clinical presentations related primarily to the sites of origin. The non-benign cases (mean diameter of 11 cases: 7.5 cm) tended to be larger than the benign cases (mean diameter of 24 cases: 4.54 cm); the statistical significance of the difference (p = 0.022) is maintained even when the outlier effect of the 1 case with a 20 cm diameter tumor reported by Folpe *et al *[14] in the non-benign group is removed. Morphologic features in the benign and non-benign cases are compared below.

## Discussion

We have presented herein the first documented case of a PEComa NOS of the uterine cervix. In addition to the primary tumor, aggregates of HMB-45+ clear cells were present at several other intraabdominal sites, including the small bowel lamina propria, ovarian hila and myometrium. Based on this pattern of distribution of tumor cells, we propose the designation "PEComatosis" to describe such aggregates. The morphogenetic basis for the intraabdominal lesions remains unclear. The similarity in morphologic features and immunophenotype between the cervical and extracervical lesions suggests that either a) both lesions arise from the same site or progenitor, or b) they both represent tissue responses of different degrees to the same stimulus. Several possibilities were considered, all of which are necessarily speculative. The lesions may be a reflection of tumor multicentricity, in which each may be a potential nidus for the development of future more well-developed tumors. Alternatively, they may be a manifestation of a poorly understood "field effect", in which there is an increased propensity to develop tumors of this type throughout the abdomen. Finally, and least likely in our opinion, they represented the tumor spread   from its primary site.

The diffuse occurrence of apparently heterologous tissue is a well-known phenomenon in the peritoneal cavity. These include *leiomyomatosis peritonealis disseminata *(LPD), *gliomatosis peritonei *and the recently described diffuse *cartilaginous metaplasia *of the peritoneum [50,52]. In LPD, for example, diffusely distributed peritoneal nodules of benign smooth muscle may proliferate or regress based on the hormonal milieu of pregnancy [52]. In this instance, circulating hormone levels are thought to represent the main stimuli underlying the proliferation of the peritoneal lesions. Even though a uterine smooth muscle tumor is associated with most cases, they are not thought to be the origin of LPD [52]. In contrast, *gliomatosis peritonei *is believed to represent an overgrowth of glial implants from ovarian teratomas [52]. In either instance, the presence of diffuse intraperitoneal lesions associated with these benign lesions (an ovarian teratoma and a uterine leiomyoma) is deemed insufficient to assign them a malignancy status [52]. These examples are directly relevant to our case, in which a malignant potential has to be assigned to a pathologically benign tumor that is showing similar appearing cells remote from its primary site. In the current case, the distinctive tropism of tumor cells for vascular structures without true intraluminal foci, argues against a hematogenous spread of tumor and argues for a *de novo *proliferation of PEC at those sites. However, whether these are foci that would have undergone involution or continued proliferating into tumor masses remains unclear. Do these lesions represent a manifestation of a poorly understood "field effect", in which there is an increased propensity to develop tumors of this type throughout abdomen? The most obvious underlying condition in this particular patient is tuberous sclerosis. A potentially comparable condition may exist in the lungs. In a few patients with and without tuberous sclerosis, a distinctive diffuse pulmonary interstitial proliferation has been described [53-56]. These proliferations are composed of clear HMB-45+ cells and are inconstantly associated with LAM [50-53]. Whether a similar mechanism is operational here is not clear. Although we do not believe the extracervical lesions represent metastases, it should be noted that contrary to the conventional paradigm, molecular evidence is accumulating regarding the "metastasis of benign tumor cells" in patients with tuberous sclerosis, although this is currently limited to the renal angiomyolipoma to pulmonary lymphangioleiomyomatosis model [57].

The issues raised by this case highlight the absence of well-established morphologic criteria predicting aggressiveness or malignancy in PEComas. Based on an analysis of 31 of the 51 reported cases detailed in Additional file 1, the following information is stated in the WHO monograph regarding the aforementioned criteria [13]: "it appears that PEComas displaying any combination of infiltrative growth, marked hypercellularity, nuclear enlargement and hyperchromasia, high mitotic activity, atypical mitotic figures, and coagulative necrosis should be regarded as malignant". However, while the presence of all the mentioned features would probably assign malignancy to *any *tumor, it is unclear what significance there is of the presence or absence of individual features or small combinations thereof.

Since the relevance of any set of pathologic criteria is ultimately dependent on their correlation to clinical behavior based on published experience, we analyzed in greater detail the clinicopathologic features of those 12 cases in which aggressive behavior was already manifest and compared them to those of the 25 cases with benign outcomes. It is well recognized by the authors that this separation is artificial, the definitional threshold for 'aggressiveness" is low, and that for example the recurrence of a tumor is by no means necessarily indicative of its malignancy. Nonetheless, this separation allows comparative analysis of groups of cases whose clinical behaviors have been shown to be different.

With regard to morphologic appearance, some features appeared to distinguish the two groups. Nuclear atypia (nucleomegaly, multinucleation, pleomorphism etc) was more likely to be present in the non-benign group, with this feature described in 8 of the 10 cases in which a comment was rendered. However, some degree of nuclear atypia was also described in 9 of the 25 cases in the benign group; the atypia in all of the latter cases were described as "minimal" or mild to moderate. Two cases that, in our opinion, showed the highest degree of nuclear atypia (in addition to high mitotic activity and necrosis) were unfortunately reported without follow-up information [10,34].

Mitotic activity was uniformly low in the benign group, with no mitotic figures identified in 13/25 (52%) cases and rare (<1/20HPF) mitotic figures found in the remainder with information. However, for the purposes of answering the more clinically relevant question, i.e. segregation of the non-benign group, mitotic activity was not useful. Only 3 of the 10 non-benign cases (in which mitotic activity information was given) showed significant mitotic activity (≥ 6/10HPF). In the remaining 7 cases, mitotic figures were described as "rare" (n = 3), "low" (n = 2) and <1/20hpF (n = 2).

Necrosis was a common feature of cases in the non-benign group, being present in 7 of 11 cases (64%), with an 8^th ^case described as showing "gelatinous-appearing material" macroscopically [11]. In contrast, of the benign group, necrosis was present in only 4 of the 18 (22%) cases in which such information was stated. Additionally, the necrosis in one of those 4 cases was described as "infarct-type" (non-coagulative) [40]. However, one of the benign cases was described as showing "scattered foci of coagulative necrosis and hemorrhage" [8]. As can be anticipated, lymphovascular invasion (LVI) by tumor was more characteristic of tumors in the non-benign group as compared to their benign counterparts: LVI was present in 4 out of 8 (50%) cases in the non-benign group and in only one case in the benign group. The latter is Case 3 of the "abdominopelvic sarcomas" described by Bonetti *et al *[6]; this tumor was a 2.5 cm well-circumscribed pelvic nodule, the patient showed no evidence of tumor recurrence or metastases at 6 months follow-up. For the purposes of this analysis, we placed this case in the benign group, definitionally based on the benign follow-up.

We also analyzed the degree and types of tumor infiltration as another potential discriminator between the benign and non-benign groups. In what remains the largest series of PEComa NOS reported to date, Vang and Kempson [40] divided 8 uterine PEComas into 2 groups (A and B) based on, in part, the degree of tumor infiltration. Of the group A, 75% of tumors showed a tongue-like myometrial infiltration reminiscent of low-grade endometrial stromal sarcoma while this type of infiltration was only focal in 75% of their group B tumors; all cases were limited to the uterus. Due to the absence of follow-up in 75% of their group A cases, the prognostic significance of this classification is unclear. Two of the four "hyalinized uterine mesenchymal neoplasms with HMB-45-positive epithelioid cells" reported by Michal and Zamecnik [25] showed a similar "tongue-like" infiltration and had a benign follow-up. All of the 7 cases of CCMMT reported by Folpe *et al *[14] "appeared circumscribed but displayed an infiltrative pattern microscopically at the periphery", a pattern remarkably similar to our case. One of their 6 cases with follow-up showed evidence of pulmonary metastases at 3 months, while follow-up was unremarkable in others. This patient reportedly died of other causes. Analysis of the usefulness of infiltration was hampered somewhat by the absence of a comment on this subject in some of the non-benign cases; however, it is unlikely to be a criterion of significant use in isolation. Only in 3 of 10 malignant cases infiltration was prominent. In 4 of the 7 remaining cases in the non-benign group, infiltration was not specifically noted; the latter includes a well-circumscribed and *encapsulated *9 cm mass involving the terminal ileum and cecum [6] which metastasized to the liver and the patient died in 28 months. The 7^th ^and 8^th ^cases showed only local infiltration [11,14]. Another case reported by Ruco *et al *[34], which we excluded from our analysis of the non-benign cases due to an absence of outcome information, consisted of a partially necrotic 5 cm mass showing high mitotic activity (11 m/10hpF) and was described as "poorly circumscribed". One of the cases described by Fukunaga [8], which we have placed in the benign group showed focal infiltrative growth. For the rest of the benign group, significant infiltration was not described in any case.

Overall, the experience with PEComas NOS is currently too limited to make a definitive assessment of prognostic features. In addition, the above analysis presumes a biologic homogeneity to tumors arising from various anatomic sites (table 2). Nonetheless, from our analysis of the reported cases with clinical outcomes as end-points, necrosis and large tumor size (both of which were more characteristic of tumors in the non-benign group) appears to have the greatest discriminatory value. However, it is likely that when more cases are described, combinations of features will provide the greatest prognostic information. In the present case, as previously noted, there was no necrosis, mitotic activity or significant pleomorphism, but there were tumor aggregates in the ovaries (perivascular) myometrium, and small bowel. The fact that close surveillance of our patient for 29 months has revealed no evidence of tumor recurrence or metastases is suggestive of tumor benignancy, although even that statement is tempered by the cases of metastases developing after long periods, up to 7 years in one case ([11], Additional file 1).

**Table 2:** Summary of pathologic features in reported cases of PEComa NOS

Pathologic feature*	**Non-Benign cases (n = 12)**	**Benign cases (n = 25)**
Cytologic atypia	8/10 (80%)	9/25 (36%)
Necrosis	7/11 (64%)	4/18 (22%)
Lymphovascular invasion	4/8 (50%)	1/11 (9.1%)
Size (average diameters)	7.5 cm (n = 11)	4.54 cm (n = 24)
Significant mitotic activity (6 mitoses per 10 high power fields or greater	3/10 (30%)	0/24 (0%)

* For each histological feature, the denominator represents the number of cases in which the information assessed was available in the published reports. See text for caveats, exceptions and more detailed analysis

An important differential consideration for PEComas arising in the uterus is epithelioid smooth muscle tumors. Vang and Kempson [40] expressed the opinion that PEComas and epithelioid smooth muscle tumors "exist on a morphologic spectrum" and recommended that HMB-45 staining be performed on all epithelioid tumors of the uterus. The contrary view was expressed by Silva *et al *[58] who demonstrated immunoreactivity for HMB-45 in 4 (80%) out of 5 "unequivocal uterine leiomyosarcomas" with epithelioid features. They concluded that HMB-45 immunoreactivity is insufficient to designate these tumors as PEComas and separate them from epithelioid smooth muscle tumors. Zamecnik and Michal [59] found immunoreactivity for HMB-45 in four distinctively hyalinized epithelioid mesenchymal tumors of the uterus, but all four cases were negative for the other three melanogenesis markers tested (Melan-A, tyrosinase and micropthalmia transcription factor). The authors concluded that their cases were closer linked to epithelioid smooth muscle tumors than PEComas. In the report by Ruco *et al *[34], twelve uterine leiomyomas, two of which were epithelioid, were negative for HMB-45. In contrast, at least focal HMB-45 positivity was demonstrated in 43 of 79 (54%) typical (non-epithelioid) smooth muscle tumors of the uterus in 2 combined series [60,61]. These somewhat contradictory findings illustrate that at this time, the relationship between epithelioid smooth muscle tumors and PEComas (outside of their shared co-expression of muscular markers) is unclear. However, since both tumors are rare, we agree with Vang and Kempson [40] that HMB-45 immunostaining should be performed on all epithelioid uterine tumors, not only to better delineate the features of both epithelioid smooth muscle tumors and PEComas, but due to the possibility of an association between the latter and the tuberous sclerosis complex (TSC).

Although the association between some members of the PEComa family (AML and LAM) and the tuberous sclerosis complex is well-known, this case represents only the third case of a PEComa NOS reported to occur in a patient with stigmata of this complex. Both of the previous cases were primary in the uterus [6,40]. Even this seemingly low rate of association (6%; 3/50) is almost certainly higher than that associated with most tumors, and may thus warrant an investigation for features of TSC in patients in whom these tumors are diagnosed.

The validity of segregating a tumor group based almost entirely on the clear appearance of constituent cells and immunoreactivity for melanogenesis markers may be proven if recurrent molecular or cytogenetic abnormalities are identified in this group. However, remarkably sparse information exists on the cytogenetic or molecular pathogenesis of PEComa NOS. Using conventional cytogenetics, Folpe *et al *[14] identified loss of X chromosome and a t(3;10) in 1 of 5 metaphases examined from a case of CCMMT. RT-PCR analysis of one perivascular epithelioid cell tumor has failed to show the EWS/ATF-1 fusion transcript from the t(12;22) characteristic of clear cell sarcoma of soft parts – another differential consideration [41]. No other cytogenetic analyses of PEComa NOS have been reported to our knowledge. p53 does not appear to be involved in the pathogenesis of these tumors, as neither p53 mutations as determined by single-stranded conformational pleomorphism analysis, nor protein overexpression as determined by immunohistochemistry have been identified [33]. Our case represents the first PEComa NOS that has been studied by CGH. Although this needs to be confirmed with more cases, the absence of chromosomal gain or loss detectable by this method, in additional to a diploid DNA content of our case, suggests that karyotypical changes may not be features of PEComa NOS.

In summary, we have documented herein the first case of a PEComa NOS of the uterine cervix occurring in a tuberous sclerosis patient. With the description of additional cases, more insight into their behavior and predictive morphologic parameters may be achieved.

## Competing Interests

The authors declare that they have no competing interests.

## Authors' contributions

OF wrote the original version of the manuscript.

PH and VP diagnosed the case and supervised the entire project.

DH and MRM collected clinical and pathologic data and participated in manuscript preparation.

DH also contributed statistical analysis.

LM, YY, PH and MBQ performed and/or analyzed and interpreted the CGH. All authors have read and approved the final manuscript.

## Additional files

Additional file 1: PEComa additional file 1. doc : All reported cases of PEComa NOS

Additional file 2: PEComa additional file 2. doc: Morphologic analysis of the 37 cases of PEComa NOS with adequate follow-up information, classified by outcome

## Supplementary Material

Additional file 1 Click here for file

Additional file 2 Click here for file
