# Correction: Perivascular epithelioid cell tumor (PEComa) of the uterine cervix associated with intraabdominal "PEComatosis": A clinicopathological study with comparative genomic hybridization analysis

**DOI:** 10.1186/1477-7819-3-25

**Published:** 2005-05-03

**Authors:** Oluwole Fadare, Vinita Parkash, Yesim Yilmaz, M Rajan Mariappan, Linglei Ma, Denise Hileeto, Mazin B Qumsiyeh, Pei Hui

**Affiliations:** 1 Department of Pathology, Yale University School of Medicine, New Haven, CT, USA; 2 Department of Laboratory Medicine Yale University School of Medicine, New Haven, CT, USA; 3 Department of Genetics, Yale University School of Medicine, New Haven, CT, USA; 4 Department of Pathology, Hospital of St Raphael, New Haven, CT, USA; 5 Department of Pediatrics, Louisiana State University Health Sciences Center, New Orleans, LA, USA; 6 Department of Pathology, Stanford University, Stanford, CA, USA; 7 Department of Pathology, New York University, New York, NY, USA

## Correction

In the original publication [1], most of the table rows in "Additional file 1" were mistakenly omitted. These files have now been converted to tables 1 and 2 in Additional file 1 in this correction article. All references in the tables refer to references in the original publication [1].

## Supplementary Material

Additional File 1 Additional file containing corrected tables 1 and 2 Click here for file

## References

[B1] Fadare O Parkash V Yilmaz Y Mariappan MR Ma L Hileeto D Qumsiyeh MB Hui P Perivascular epithelioid cell tumor (PEComa) of the uterine cervix associated with intraabdominal "PEComatosis": A clinicopathological study with comparative genomic hybridization analysis World J Surg Oncol 2004 2 35 15494070 10.1186/1477-7819-2-35 PMC527874

