# Supplementary material for: Perivascular epithelioid cell tumor (PEComa) of the uterine cervix associated with intraabdominal "PEComatosis": A clinicopathological study with comparative genomic hybridization analysis
Source: World J Surg Oncol. 2004 Oct 19;2:35. doi: 10.1186/1477-7819-2-35 (PMC527874; doi:10.1186/1477-7819-2-35)
Supplement: Additional file 1 [file 1477-7819-2-35-S1.doc]

**Additional file 1: All reported cases of PEComa NOS**

|  | **Reference** | **Year** | **Primary site** | **Age/Sex** | **Size** | **Authors' designation** | **Outcome** | **TSC** |
| --- | --- | --- | --- | --- | --- | --- | --- | --- |
| 1 | Adachi et al [1] | 2004 | Kidney | 71/F | 3cm | PECT | NERM at 76 months | No |
| 2 | Sadeghi et al [43] | 2004 | Common bile duct | 51/M | 2cm | PECT | RECENT | Ns |
| 3 | Bhalla et al [44] | 2004 | Kidneys (multiple) | 70/F | 11cm | PECT (Pecoma) | NERM 36 months | No |
| 4 | Fukunaga [8] | 2004 | Uterus | 32/F | 5cm | PECT | NERM 8 months | No |
| 5 | Lehman [22] | 2004 | Skull base | 49/F | 5cm | PEComa | Death at 6 weeks | No |
| 6 | Fink et al [45] | 2004 | Broad ligament | 51/F | 17cm | PECT (Pecoma) | NERM at 15 months | No |
| 7 | Gao et al [2004] | 2004 | Uterus | 60/F | 4cm | PECT (Pecoma) | NS | No |
| 8 | Fukunaga [12] | 2004 | Soft tissue (abd. wall) | 44/F | 3.5cm | PECT (Pecoma) | Recurrence at 6 years | No |
| 9 | Crowson et al [7] | 2003 | Skin (scalp) | 58/M | 0.8cm | CCMMT-PECT | NS | Ns |
| 10 | Yanai et al [41] | 2003 | Jejunum | 32/F | 7.5cm | PECT | Recurrence after 13 months | No |
| 11 | Diment/Colecchia [10] | 2003 | Soft tissue (thigh) | 59/F | 10cm | Myomelanocytic tumor | NS | No |
| 12 | Dimmler et al [11] | 2003 | Uterus(Subserosa) | 61/F | 4cm | PEComa | Metastases at 7 years | Ns |
| 13 | Greene et al [19] | 2003 | Uterus | 79/F | 13cm | PEComa | Death at 2+ years, mets | Ns |
| 14 | Park et al [31] | 2003 | Uterus | 32/F | 8.0cm | PEComa | NERM at 18 months | No |
| 15 | Pan et al [27] | 2003 | Urinary Bladder | 33/F | 4.0cm | CCMMT | NERM at 6 years | No |
| 16 | Pan et al [28] | 2003 | Prostate | 46/M | 8.5cm | PEComa | Death at 4 years, mets | No |
| 17 | Folpe et al [15] | 2002 | Soft tissue (thigh) | 43/F | 3.5cm | CCMMT | RECENT | No |
| 18 | Manganaro et al [24] | 2002 | Pelvic | 56/F | "large" | PEComa | Recurrence after 4 years | Ns |
| 19 | Govender et al [20] | 2002 | Breast | 16/F | 6cm | Clear cell "sugar" tumor | NERM at 9 months | No |
| 20 | Vang/Kempson [40] | 2002 | Uterus | 40/F | 12cm | PEComa | Unavailable | No |
| 21 |  | 2002 | Uterus | 54/F | 0.6cm | PEComa | RECENT | No |
| 22 |  | 2002 | Uterus | 56/F | 1cm | PEComa | RECENT | No |
| 23 |  | 2002 | Uterus | 75/F | 5cm | PEComa | NERM at 2.6 years | No |
| 24 |  | 2002 | Uterus | 47/F | 4.5cm | PEComa | NERM at 6 weeks | No |
| 25 |  | 2002 | Uterus | 49/F | 4.0,2.5cm | PEComa | NERM at 4.5 years | Yes |
| 26 |  | 2002 | Uterus | 55/F | 4.5cm | PEComa | NERM at 2 months | No |
| 27 |  | 2002 | Uterus | 58/F | 1.5cm | PEComa | Unavailable | No |
| 28 | Tazelaar et al [38] | 2001 | Rectum | 9/F | 3.0cm | PEST | NERM at 14 months | Ns |
| 29 |  | 2001 | Perineum | 20/F | 2cm | PEST | NERM at 4 years | Ns |
| 30 |  | 2001 | Heart (atrium) | 29/M | NS | PEST | Death from coronary thrombosis | Ns |
| 31 |  | 2001 | Rectum | 40/F | NS | PEST | NERM at 6 months | Ns |
| 32 | Bonetti et al [6] | 2001 | Cecum/terminal ileum | 28/F | 9cm | ABD of PEC | Death at 28 months, mets | No |
| 33 |  | 2001 | Uterus | 19/F | 5.5cm | ABD of PEC | Metastases at 18 months | No |
| 34 |  | 2001 | Pelvic | 40/F | 2.5cm | ABD of PEC | NERM at 6 months | Unkn |
| 35 |  | 2001 | Uterus | 41/F | 6cm | ABD of PEC | NERM at 6 months | Yes |
| 36 | Folpe et al [14] | 2000 | Ligamentum teres/FL | 29/M | 20cm | CCMMT | Metastases at 3 months | No |
| 37 |  | 2000 | Ligamentum teres/FL | 11/F | 9cm | CCMMT | NERM At 5 years | No |
| 38 |  | 2000 | Ligamentum teres/FL | 21/F | 8.5cm | CCMMT | NERM at 2 years | No |
| 39 |  | 2000 | Ligamentum teres/FL | 10/F | 5cm | CCMMT | Unavailable | No |
| 40 |  | 2000 | Ligamentum teres/FL | 6/F | 5cm | CCMMT | NERM at 2 years | No |
| 41 |  | 2000 | Ligamentum teres/FL | 3/F | 5.5cm | CCMMT | NERM at 10 months | No |
| 42 |  | 2000 | Omentum | 15/F | 8cm | CCMMT | NERM at 6 months | No |
| 43 | Tanaka et al [37] | 2000 | Ligamentum teres | 13/F | 9cm | Clear cell "sugar" tumor | NERM at 22 years | No |
| 44 | Michal/Zamecnik [25] | 2000 | Uterus | 58/F | 2cm | HUMN with HMB-45 EC | NERM at 4 years | No |
| 45 |  | 2000 | Uterus | 48/F | 7cm | HUMN with HMB-45 EC | NERM at 4 years | No |
| 46 |  | 2000 | Uterus | 46/F | 1.5cm | HUMN with HMB-45 EC | NERM at 1 year | No |
| 47 |  | 2000 | Uterus | 46/F | 2.5cm | HUMN with HMB-45 EC | NERM at 1 year | No |
| 48 | Ruco et al [34] | 1998 | Uterus | 56/F | 5cm | Epithelioid LAM-like tumor | Not stated | No |
| 49 | Pea et al [32] | 1996 | Uterus | 57/F | 2cm | Composed of PEC | NERM at 2 years | No |
| 50 | Zamboni et al [42] | 1996 | Pancreas | 60/F | 2cm | Clear cell "sugar" tumor | NERM at 3 months | No |
| 51 | Kung et al [23] | 1984 | Trachea | 48/F | 2.5cm | Clear cell "sugar" tumor | NERM at 6 years | Ns |

*1) Excludes all variants of AML including monotypic forms reported under such appellations as renal capsuloma, renal epithelioid oxyphillic neoplasms or monotypic epithelioid angiomyolipoma. 2) Excludes the case(s) of abdominopelvic sarcoma reported by Panizo et al/Panizo-Santos et al [29,30] and Sola et al [36] due to lack of clinicopathologic information. 3) Presumes the same case is being reported in Ruco et al [34] and D’Andrea et al [9].

NERM: No evidence of recurrence or metastases; ABDS: Abdominopelvic sarcoma; ABD: Abdominal; PEST: Primary extrapulmonary sugar tumor; CMMMT: clear cell myomelanocytic tumor; FL: falciform ligament; CCST: clear cell sugar tumor of the lung; AML: angiomyolipoma; PECT: perivascular epithelioid cell tumor; LAM: lymphangioleiomyomatosis; NS: Not specifically stated; TSC: tuberous sclerosis complex; HUMN WITH HMB-45 EC: Hyalinized uterine mesenchymal neoplasms with HMB-45 epithelioid cells; UNKN: unknown; METS: metastases
