# Supplementary material for: Perivascular epithelioid cell tumor (PEComa) of the uterine cervix associated with intraabdominal "PEComatosis": A clinicopathological study with comparative genomic hybridization analysis
Source: World J Surg Oncol. 2004 Oct 19;2:35. doi: 10.1186/1477-7819-2-35 (PMC527874; doi:10.1186/1477-7819-2-35)
Supplement: Additional file 2 [file 1477-7819-2-35-S2.doc]

Additional file 2: Morphologic analysis of the 37 cases of PEComa NOS with adequate follow-up information, classified by outcome.

.

|  | **Reference** | | **Primary site** | | **Size** | **Atypia** | **Mitoses** | **Necrosis** | **Infiltrative** | **LVI** | | **Follow-up** | |
| --- | --- | --- | --- | --- | --- | --- | --- | --- | --- | --- | --- | --- | --- |
| ***non-benign cases*** | | | | | | | | | | | | | |
| 1 | Yanai et al [41] | | Jejunum | | 7.5cm | Yes | NS | Yes | No | No | | Recurrence of tumor at  13 months (pelvic side wall)  with ovarian metastases  at 25 months | |
| 2 | Dimmler et al [11] | | Uterus (subserosa) | | 4cm | No | "Low" | Gelatinous^ | Local | Yes | | Pulmonary  metastases  (2 cm and 0.3cm)  at 7 years after  original resection | |
| 3 | Greene et al [19] | | Uterus | | 13cm | Yes | 8/10HPF | Yes | NS | NS | | Death at 2+years  following recurrence  in pelvic sidewall and  colonic mesentery | |
| 4 | Pan et al[27] | | Prostate | | 8.5cm | Yes | "Low" | Yes | No | No | | Death at 4 years  following pulmonary  metastases at 3 years | |
| 5 | Manganaro et al [24] | | Pelvic | | "large" | NS | NS | NS | NS | NS | | Recurrence of tumor  at 4 years (sizes  of recurrences  7 cm and 5cm) | |
| 6 | Bonetti et al [6] | | Terminal ileum serosa | | 9cm | Yes | "Rare" | Yes | No | Yes | | Death at 28 months  with hepatic metastases | |
| 7 | Bonetti et al [6] | | Lower uterine segment | | 5.5cm | Yes | "Rare" | Yes | Yes | Yes | | Pulmonary and bone  metastases at 18 months;  Lost to follow-up thereafter | |
| 8 | Bonetti et al [6] | | Uterus | | 6cm | Yes | "Rare" | Yes | No | Yes | | Metastases to  Ovary at presentation.  NERM at 6 months | |
| 9 | Folpe et al [14] | | Ligamentum teres/FL | | 20cm | No | <1/20HPF | No | No^¶^ | No | | Radiographic evidence  of pulmonary metastases  at 3 months. Death from  other causes (1yr) | |
| 10 | Park et al [31] | | Uterus | | 8cm | NS | <1/50HPF | Yes | Yes | NS | | Metastases to  mesovarium &  mesosalpinx  at presentation.  NERM at 18 months | |
| 11 | Fukunaga [8] | | Soft tissue (abd wall) | | 3.5cm | Yes | 6/10HPF | No | No | No | | Recurrence at  6 years; NERM  for 2 years  thereafter | |
| 12 | Lehman [22] | | Skull base | | 5cm | Yes | 3/1HPF(400X) | No | Yes | NS | | Death at 6 weeks  with paraspinal  spread and  probable  pulmonary  metastases | |
| ***Benign cases*** | | | | | | | | | | | | | |
| 1 | Adachi et al [1] | Kidney | | 3cm | | minimal | NS | NS | NO | | NS | | NERM at 76 months |
| 2 | Pan et al [27] | Urinary Bladder | | 4cm | | NO | NONE | NO | NO | | NS | | NERM at 72 months |
| 3 | Govender et al [20] | Breast | | 6cm | | minimal | NONE | YES | NO | | NS | | NERM at 9 months |
| 4 | Vang & Kempson **^Ж^**Φ[40] | Uterus | | 5cm | | NO | NONE | NO | NO | | NS | | NERM at 31.2 months |
| 5 |  | Uterus | | 4.5cm | | YES | NONE | NO | NO | | NS | | NERM at 1.5 months |
| 6 |  | Uterus | | 4cm | | YES | NONE | NONE | NO | | NS | | NERM at 54 months |
| 7 |  | Uterus | | 4.5cm | | NO | NONE | Infarct-type | NO | | NS | | NERM at 2 months |
| 8 | Tazelaar et al [38] | Rectum | | 3cm | | NO | RARE | NO | NO | | NO | | NERM at 14 months |
| 9 |  | Rectum | | NS | | NO | RARE | NO | NO | | NO | | NERM at 6 months |
| 10 |  | Perineum | | 2cm | | NO | RARE | NO | NO | | NO | | NERM at 48 months |
| 11 | Bonetti et al [6] | Pelvic | | 2.5cm | | YES | RARE | YES | NO | | YES | | NERM at 6 months |
| 12 | Folpe al et al [14] | Ligamentum teres/FL | | 9cm | | NO | <1/20hPF | NO | NO^¶^ | | NO | | NERM at 60 months |
| 13 |  | Ligamentum teres/FL | | 8.5cm | | NO | <1/20hPF | NO | NO^¶^ | | NO | | NERM at 24 months |
| 14 |  | Ligamentum teres/FL | | 5cm | | NO | <1/20hPF | NO | NO^¶^ | | NO | | NERM at 24 months |
| 15 |  | Ligamentum teres/FL | | 5.5cm | | NO | <1/20hPF | NO | NO^¶^ | | NO | | NERM at 10 months |
| 16 |  | Omentum | | 8cm | | NO | <1/20hPF | NO | NO^¶^ | | NO | | NERM at 6 months |
| 17 | Tanaka et al [37] | Ligamentum teres | | 9cm | | YES | NONE | NO | NO | | NO | | NERM at 264 months |
| 18 | Michal & Zamecnik**^Ж^**#[25] | Uterus | | 2cm | | NO | NONE | NS | NO | | NS | | NERM at 48 months |
| 19 |  | Uterus | | 7cm | | NO | NONE | NS | NO | | NS | | NERM at 48 months |
| 20 |  | Uterus | | 1.5cm | | NO | NONE | NS | NO | | NS | | NERM at 12 months |
| 21 |  | Uterus | | 2.5cm | | NO | NONE | NS | NO | | NS | | NERM at 12 months |
| 22 | Pea et al [32] | Uterus | | 2cm | | minimal | NONE | NS | NO | | NS | | NERM at 24 months |
| 23 | Zamboni et al [42] | Pancreas | | 2cm | | YES | NONE | NS | NO | | NS | | NERM at 3 months |
| 24 | Kung, et al [23] | Trachea | | 2.5cm | | NO | NONE | NS | NO | | NS | | NERM at 72 months |
| 25 | Fukunaga [8] | Uterus | | 5cm | | Mild | 5/50 hPf | YES | Focal | | NO | | NERM at 8 months |

NERM: No evidence of recurrence or metastases; NS: Not specifically stated; ABD: Abdominal; LVI: Lymphovascular invasion; HPF: High power field; FL: Falciform ligament;¶ Microscopic infiltration at periphery ;Ж Organ-confined tongue-like infiltration present in some cases; # Subendothelial pattern of tumor growth in some cases; Φ Intravascular leiomyomatosis-like vascular invasion pattern in one case.; Macroscopic: ^gelatinous appearing material
