# Supplementary material for: Correction: Perivascular epithelioid cell tumor (PEComa) of the uterine cervix associated with intraabdominal "PEComatosis": A clinicopathological study with comparative genomic hybridization analysis
Source: World J Surg Oncol. 2005 May 3;3:25. doi: 10.1186/1477-7819-3-25 (PMC1097764; doi:10.1186/1477-7819-3-25)
Supplement: Additional File 1 — Additional file containing corrected tables 1 and 2 [file 1477-7819-3-25-S1.doc]

| Table 1: All Reported Cases of PEComa NOS | | | | | | | | |
| --- | --- | --- | --- | --- | --- | --- | --- | --- |
|  | **Reference** | **Year** | **Primary site** | **Age/sex** | **Size** | **Authors' designation** | **Outcome** | **TSC** |
| 1 | Adachi et al [1] | 2004 | Kidney | 71/F | 3cm | PECT | NERM at 76 months | NO |
| 2 | Sadeghi et al [43] | 2004 | Common bile duct | 51/M | 2cm | PECT | RECENT | NS |
| 3 | Bhalla et al [44] | 2004 | Kidneys (multiple) | 70/F | 11cm | PECT (Pecoma) | NERM 36 months | NO |
| 4 | Fukunaga [8] | 2004 | Uterus | 32/F | 5cm | PECT | NERM 8 months | NO |
| 5 | Lehman [22] | 2004 | Skull base | 49/F | 5cm | PEComa | Death at 6 weeks | NO |
| 6 | Fink et al [45] | 2004 | Broad ligament | 51/F | 17cm | PECT (Pecoma) | NERM at 15 months | NO |
| 7 | Gao et al [46] | 2004 | Uterus | 60/F | 4cm | PECT (Pecoma) | NS | NO |
| 8 | Fukunaga [12] | 2004 | Soft tissue (abd. wall) | 44/F | 3.5cm | PECT (Pecoma) | Recurrence at 6 years | NO |
| 9 | Crowson et al [7] | 2003 | Skin (scalp) | 58/M | 0.8cm | CCMMT-PECT | NS | NS |
| 10 | Yanai et al [41] | 2003 | Jejunum | 32/F | 7.5cm | PECT | Recurrence after 13 months | NO |
| 11 | Diment/Colecchia [10] | 2003 | Soft tissue (thigh) | 59/F | 10cm | Myomelanocytic tumor | NS | NO |
| 12 | Dimmler et al [11] | 2003 | Uterus(Subserosa) | 61/F | 4cm | PEComa | Metastases at 7 years | NS |
| 13 | Greene et al [19] | 2003 | Uterus | 79/F | 13cm | PEComa | Death at 2+ years, mets | NS |
| 14 | Park et al [31] | 2003 | Uterus | 32/F | 8.0cm | PEComa | NERM at 18 months | NO |
| 15 | Pan et al [27] | 2003 | Urinary Bladder | 33/F | 4.0cm | CCMMT | NERM at 6 years | NO |
| 16 | Pan et al [28] | 2003 | Prostate | 46/M | 8.5cm | PEComa | Death at 4 years, mets | NO |
| 17 | Folpe et al [15] | 2002 | Soft tissue (thigh) | 43/F | 3.5cm | CCMMT | RECENT | NO |
| 18 | Manganaro et al [24] | 2002 | Pelvic | 56/F | "large" | PEComa | Recurrence after 4 years | NS |
| 19 | Govender et al [20] | 2002 | Breast | 16/F | 6cm | Clear cell "sugar" tumor | NERM at 9 months | NO |
| 20 | Vang/Kempson [40] | 2002 | Uterus | 40/F | 12cm | PEComa | Unavailable | NO |
| 21 | Vang/Kempson [40] | 2002 | Uterus | 54/F | 0.6cm | PEComa | RECENT | NO |
| 22 | Vang/Kempson [40] | 2002 | Uterus | 56/F | 1cm | PEComa | RECENT | NO |
| 23 | Vang/Kempson [40] | 2002 | Uterus | 75/F | 5cm | PEComa | NERM at 2.6 years | NO |
| 24 | Vang/Kempson [40] | 2002 | Uterus | 47/F | 4.5cm | PEComa | NERM at 6 weeks | NO |
| 25 | Vang/Kempson [40] | 2002 | Uterus | 49/F | 4.0,2.5cm | PEComa | NERM at 4.5 years | YES |
| 26 | Vang/Kempson [40] | 2002 | Uterus | 55/F | 4.5cm | PEComa | NERM at 2 months | NO |
| 27 | Vang/Kempson [40] | 2002 | Uterus | 58/F | 1.5cm | PEComa | Unavailable | NO |
| 28 | Tazelaar et al [38] | 2001 | Rectum | 9/F | 3.0cm | PEST | NERM at 14 months | NS |
| 29 | Tazelaar et al [38] | 2001 | Perineum | 20/F | 2cm | PEST | NERM at 4 years | NS |
| 30 | Tazelaar et al [38] | 2001 | Heart (atrium) | 29/M | NS | PEST | Death from coronary thrombosis | NS |
| 31 | Tazelaar et al [38] | 2001 | Rectum | 40/F | NS | PEST | NERM at 6 months | NS |
| 32 | Bonetti et al [6] | 2001 | Cecum/terminal ileum | 28/F | 9cm | ABD of PEC | Death at 28 months, mets | NO |
| 33 | Bonetti et al [6] | 2001 | Uterus | 19/F | 5.5cm | ABD of PEC | Metastases at 18 months | NO |
| 34 | Bonetti et al [6] | 2001 | Pelvic | 40/F | 2.5cm | ABD of PEC | NERM at 6 months | UNKN |
| 35 | Bonetti et al [6] | 2001 | Uterus | 41/F | 6cm | ABD of PEC | NERM at 6 months | YES |
| 36 | Folpe et al [14] | 2000 | Ligamentum teres/FL | 29/M | 20cm | CCMMT | Metastases at 3 months | NO |
| 37 | Folpe et al [14] | 2000 | Ligamentum teres/FL | 11/F | 9cm | CCMMT | NERM At 5 years | NO |
| 38 | Folpe et al [14] | 2000 | Ligamentum teres/FL | 21/F | 8.5cm | CCMMT | NERM at 2 years | NO |
| 39 | Folpe et al [14] | 2000 | Ligamentum teres/FL | 10/F | 5cm | CCMMT | Unavailable | NO |
| 40 | Folpe et al [14] | 2000 | Ligamentum teres/FL | 6/F | 5cm | CCMMT | NERM at 2 years | NO |
| 41 | Folpe et al [14] | 2000 | Ligamentum teres/FL | 3/F | 5.5cm | CCMMT | NERM at 10 months | NO |
| 42 | Folpe et al [14] | 2000 | Omentum | 15/F | 8cm | CCMMT | NERM at 6 months | NO |
| 43 | Tanaka et al [37] | 2000 | Ligamentum teres | 13/F | 9cm | Clear cell "sugar" tumor | NERM at 22 years | NO |
| 44 | Michal/Zamecnik [25] | 2000 | Uterus | 58/F | 2cm | HUMN with HMB-45 EC | NERM at 4 years | NO |
| 45 | Michal/Zamecnik [25] | 2000 | Uterus | 48/F | 7cm | HUMN with HMB-45 EC | NERM at 4 years | NO |
| 46 | Michal/Zamecnik [25] | 2000 | Uterus | 46/F | 1.5cm | HUMN with HMB-45 EC | NERM at 1 year | NO |
| 47 | Michal/Zamecnik [25] | 2000 | Uterus | 46/F | 2.5cm | HUMN with HMB-45 EC | NERM at 1 year | NO |
| 48 | Ruco et al [34] | 1998 | Uterus | 56/F | 5cm | Epithelioid LAM-like tumor | Not stated | NO |
| 49 | Pea et al [32] | 1996 | Uterus | 57/F | 2cm | Composed of PEC | NERM at 2 years | NO |
| 50 | Zamboni et al [42] | 1996 | Pancreas | 60/F | 2cm | Clear cell "sugar" tumor | NERM at 3 months | NO |
| 51 | Kung et al [23] | 1984 | Trachea | 48/F | 2.5cm | Clear cell "sugar" tumor | NERM at 6 years | NS |

*1) Excludes all variants of AML including monotypic forms reported under such appellations as renal capsuloma, renal epithelioid oxyphillic neoplasms or monotypic epithelioid angiomyolipoma. 2) Excludes the case(s) of abdominopelvic sarcoma reported by Panizo et al/Panizo-Santos et al [29,30] and Sola et al [36] due to lack of clinicopathologic information. 3) Presumes the same case is being reported in Ruco et al [34] and D’Andrea et al [9].

**ABBREVIATIONS**:

NERM: No evidence of recurrence or metastases; ABDS: Abdominopelvic sarcoma; ABD: Abdominal; PEST: Primary extrapulmonary sugar tumor; CMMMT: clear cell myomelanocytic tumor; FL: falciform ligament; CCST: clear cell sugar tumor of the lung; AML: angiomyolipoma; PECT: perivascular epithelioid cell tumor; LAM: lymphangioleiomyomatosis; NS: Not specifically stated; TSC: tuberous sclerosis complex; HUMN WITH HMB-45 EC: Hyalinized uterine mesenchymal neoplasms with HMB-45 epithelioid cells; UNKN: unknown; METS: metastases

| Table 2: Morphologic analysis of 37 cases of PEComa NOS with  adequate follow-up information, classified by outcome | | | | | | | | | |
| --- | --- | --- | --- | --- | --- | --- | --- | --- | --- |
|  | | | **Non-benign cases** | | |  | | | |
|  | **Reference** | **Primary site** | **Size** | **Atypia** | **Mitoses** | **Necrosis** | **Infiltrative** | **LVI** |  |
| 1 | Yanai et al [41] | Jejunum | 7.5cm | Yes | NS | Yes | No | No | Recurrence of tumor at  13 months (pelvic side wall)  with ovarian metastases  at 25 months |
| 2 | Dimmler et al [11] | Uterus (subserosa) | 4cm | No | "Low" | Gelatinous^ | Local | Yes | Pulmonary  metastases  (2 cm and 0.3cm)  at 7 years after  original resection |
| 3 | Greene et al [19] | Uterus | 13cm | Yes | 8/10HPF | Yes | NS | NS | Death at 2+years  following recurrence  in pelvic sidewall and  colonic mesentery |
| 4 | Pan et al [28] | Prostate | 8.5cm | Yes | "Low" | Yes | No | No | Death at 4 years  following pulmonary  metastases at 3 years |
| 5 | Manganaro et al [24] | Pelvic | "large" | NS | NS | NS | NS | NS | Recurrence of tumor  at 4 years (sizes  of recurrences  7 cm and 5cm) |
| 6 | Bonetti et al [6] | Terminal ileum serosa | 9cm | Yes | "Rare" | Yes | No | Yes | Death at 28 months  with hepatic metastases |
| 7 | Bonetti et al [6] | Lower uterine segment | 5.5cm | Yes | "Rare" | Yes | Yes | Yes | Pulmonary and bone  metastases at 18 months;  Lost to follow-up thereafter |
| 8 | Bonetti et al [6] | Uterus | 6cm | Yes | "Rare" | Yes | No | Yes | Metastases to  Ovary at presentation.  NERM at 6 months |
| 9 | Folpe et al [14] | Ligamentum teres/FL | 20cm | No | <1/20HPF | No | No^¶^ | No | Radiographic evidence  of pulmonary metastases  at 3 months. Death from  other causes (1yr) |
| 10 | Park et al [31] | Uterus | 8cm | NS | <1/50HPF | Yes | Yes | NS | Metastases to  mesovarium &  mesosalpinx  at presentation.  NERM at 18 months |
| 11 | Fukunaga [12] | Soft tissue (abd wall) | 3.5cm | Yes | 6/10HPF | No | No | No | Recurrence at  6 years; NERM  for 2 years  thereafter |
| 12 | Lehman [22] | Skull base | 5cm | Yes | 3/1HPF(400X) | No | Yes | NS | Death at 6 weeks  with paraspinal  spread and  probable  pulmonary  metastases |
|  | | | | | | | | | |
|  |  | | **Benign Cases** | |  | | | | |
|  | **Reference** | **Primary site** | **Size** | **Atypia** | **Mitoses** | **Necrosis** | **Infiltrative** | **LVI** | **Outcome** |
| 1 | Adachi et al [1] | Kidney | 3cm | minimal | NS | NS | NO | NS | NERM at 76 months |
| 2 | Pan et al [27] | Urinary Bladder | 4cm | NO | NONE | NO | NO | NS | NERM at 72 months |
| 3 | Govender et al [20] | Breast | 6cm | minimal | NONE | YES | NO | NS | NERM at 9 months |
| 4 | Vang & Kempson **^Ж^**Φ [40] | Uterus | 5cm | NO | NONE | NO | NO | NS | NERM at 31.2 months |
| 5 |  | Uterus | 4.5cm | YES | NONE | NO | NO | NS | NERM at 1.5 months |
| 6 |  | Uterus | 4cm | YES | NONE | NONE | NO | NS | NERM at 54 months |
| 7 |  | Uterus | 4.5cm | NO | NONE | Infarct-type | NO | NS | NERM at 2 months |
| 8 | Tazelaar et al [38] | Rectum | 3cm | NO | RARE | NO | NO | NO | NERM at 14 months |
| 9 |  | Rectum | NS | NO | RARE | NO | NO | NO | NERM at 6 months |
| 10 |  | Perineum | 2cm | NO | RARE | NO | NO | NO | NERM at 48 months |
| 11 | Bonetti et al [6] | Pelvic | 2.5cm | YES | RARE | YES | NO | YES | NERM at 6 months |
| 12 | Folpe al et al [14] | Ligamentum teres/FL | 9cm | NO | <1/20hPF | NO | NO^¶^ | NO | NERM at 60 months |
| 13 |  | Ligamentum teres/FL | 8.5cm | NO | <1/20hPF | NO | NO^¶^ | NO | NERM at 24 months |
| 14 |  | Ligamentum teres/FL | 5cm | NO | <1/20hPF | NO | NO^¶^ | NO | NERM at 24 months |
| 15 |  | Ligamentum teres/FL | 5.5cm | NO | <1/20hPF | NO | NO^¶^ | NO | NERM at 10 months |
| 16 |  | Omentum | 8cm | NO | <1/20hPF | NO | NO^¶^ | NO | NERM at 6 months |
| 17 | Tanaka et al [37] | Ligamentum teres | 9cm | YES | NONE | NO | NO | NO | NERM at 264 months |
| 18 | Michal & Zamecnik**^Ж^**# [25] | Uterus | 2cm | NO | NONE | NS | NO | NS | NERM at 48 months |
| 19 |  | Uterus | 7cm | NO | NONE | NS | NO | NS | NERM at 48 months |
| 20 |  | Uterus | 1.5cm | NO | NONE | NS | NO | NS | NERM at 12 months |
| 21 |  | Uterus | 2.5cm | NO | NONE | NS | NO | NS | NERM at 12 months |
| 22 | Pea et al [33] | Uterus | 2cm | minimal | NONE | NS | NO | NS | NERM at 24 months |
| 23 | Zamboni et al [42] | Pancreas | 2cm | YES | NONE | NS | NO | NS | NERM at 3 months |
| 24 | Kung et al [23] | Trachea | 2.5cm | NO | NONE | NS | NO | NS | NERM at 72 months |
| 25 | Fukunaga [8] | Uterus | 5cm | Mild | 5/50 hPf | YES | Focal | NO | NERM at 8 months |

**ABBREVIATIONS**:

NERM: No evidence of recurrence or metastases; NS: Not specifically stated; ABD: Abdominal; LVI: Lymphovascular invasion; HPF: High power field; FL: Falciform ligament;¶ Microscopic infiltration at periphery;Ж Organ-confined tongue-like infiltration present in some cases; # Subendothelial pattern of tumor growth in some cases; Φ Intravascular leiomyomatosis-like vascular invasion pattern in one case.; Macroscopic: ^gelatinous appearing material
